# Supplementary material for: Joint Crisis Plan in Mental Health Settings: A Reflective Process More than an Intervention Tool?
Source: Healthcare (Basel). 2024 Dec 16;12(24):2532. doi: 10.3390/healthcare12242532 (PMC11675284; doi:10.3390/healthcare12242532)

Joint crisis plan

The plan, completed in collaboration with healthcare providers and my close ones, aims to identify the triggers that may lead me toward a crisis situation, as well as the tools that help us act in prevention, during, and after the crisis.

**Current situation**
What is my main difficulty at the moment?
What are my personal goals at the moment?
**Crisis triggers and early warning signs**
What are the situations where I feel overwhelmed and that may lead me to a crisis situation?
How am I specifically when I am in a crisis situation? (i.e.: thoughts, emotions, physical reactions, behaviors)
What are the other signs that can appear according to my close ones?
**What can I do to help me?**
What are my strengths that can help when I am in a crisis situation?
What are the personal strategies that can help me calm and feel better when I feel I might lose control of my emotions and behaviors?

Version developed in French and evaluated as part of the study led by Marie-Hélène Goulet, RN, PhD, adapted from Ferrari et al. (2018). Version translated into English for publication purposes.

CC BY-NC-SA 4.0

What could others do to help me?	
When I start to lose control?	When I lose control?

What should others avoid doing to help me?	
When I start to lose control?	When I lose control?
What are the possible alternatives to the actions to avoid?	

MY PERSONAL RESOURCES
**Within my circle, if needed, I can ask help to:**
Family name, First name: ___________________________ Telephone: _____________________<br>Connection with me: ___________________<br>**In a crisis situation, I want this person to be informed by the professional team first.**
Family name, First name: ___________________________ Telephone: _____________________<br>Connection with me: ___________________

MY PROFESSIONAL RESOURCES
**Among the professionals that collaborate with me, if needed, I can ask help to:**
Family name, First name: ___________________________ Telephone: _____________________<br>Role : ___________________
Family name, First name: ___________________________ Telephone: _____________________<br>Role : ___________________

MY COMMUNITY RESOURCES
**If needed, I can also ask help to:**
• [*A list of community resources is recorded and adjusted based on the context where the joint crisis plan has been implemented.*]

This joint crisis plan was jointly written by:

______________________(user signature),

______________________(close one signature), **and**

______________________(professional signature) **on :** __________________(date).

Version developed in French and evaluated as part of the study led by Marie-Hélène Goulet, RN, PhD, adapted from Ferrari et al. (2018). Version translated into English for publication purposes. CC BY-NC-SA 4.0

Supplementary File S2

Table S1. ARIMA Model Parameters.

Parameters	Estimate	Error	*t*	*p*
AR1	1.59	0.12	12.75	<0.001
AR2	-0.81	0.12	-6.71	<0.001
MA1	1.62	0.094	17.13	<0.001
MA2	-0.91	0.088	-10.36	<0.001
Intervention	-9097.98	4260.12	-2.14	0.035

Figure S1. Autocorrelation and Partial Autocorrelation Function Plots.

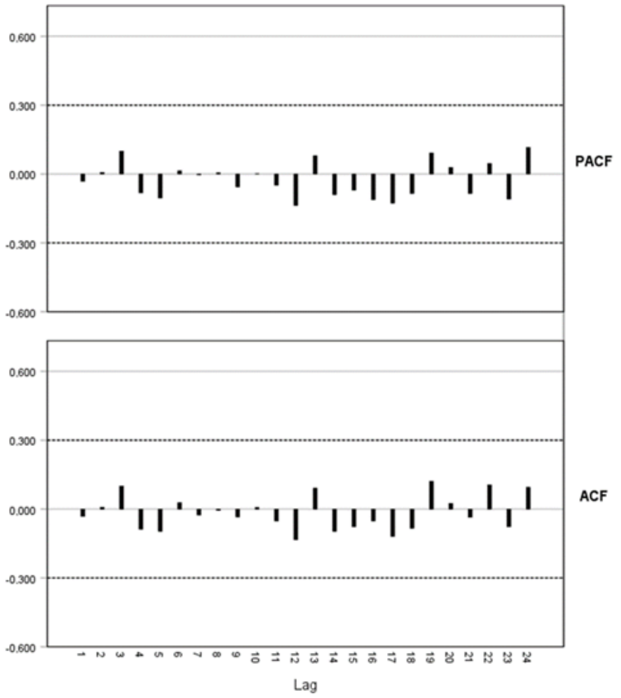

Supplement: Supplementary file 1 [file healthcare-12-02532-s001.zip › healthcare-3353139-supplementary.pdf]
